# Mitochondrial Genome Analysis Reveals Historical Lineages in Yellowstone Bison

**DOI:** 10.1371/journal.pone.0166081

**Published:** 2016-11-23

**Authors:** David Forgacs, Rick L. Wallen, Lauren K. Dobson, James N. Derr

**Affiliations:** 1 Department of Veterinary Pathobiology, Texas A&M University, College Station, Texas, United States of America; 2 National Park Service, Yellowstone National Park, Mammoth Hot Springs, Wyoming, United States of America; University of Texas Health Science Center at San Antonio, UNITED STATES

## Abstract

Yellowstone National Park is home to one of the only plains bison populations that have continuously existed on their present landscape since prehistoric times without evidence of domestic cattle introgression. Previous studies characterized the relatively high levels of nuclear genetic diversity in these bison, but little is known about their mitochondrial haplotype diversity. This study assessed mitochondrial genomes from 25 randomly selected Yellowstone bison and found 10 different mitochondrial haplotypes with a haplotype diversity of 0.78 (± 0.06). Spatial analysis of these mitochondrial DNA (mtDNA) haplotypes did not detect geographic population subdivision (F_ST_ = -0.06, p = 0.76). However, we identified two independent and historically important lineages in Yellowstone bison by combining data from 65 bison (defined by 120 polymorphic sites) from across North America representing a total of 30 different mitochondrial DNA haplotypes. Mitochondrial DNA haplotypes from one of the Yellowstone lineages represent descendants of the 22 indigenous bison remaining in central Yellowstone in 1902. The other mitochondrial DNA lineage represents descendants of the 18 females introduced from northern Montana in 1902 to supplement the indigenous bison population and develop a new breeding herd in the northern region of the park. Comparing modern and historical mitochondrial DNA diversity in Yellowstone bison helps uncover a historical context of park restoration efforts during the early 1900s, provides evidence against a hypothesized mitochondrial disease in bison, and reveals the signature of recent hybridization between American plains bison (*Bison bison bison*) and Canadian wood bison (*B*. *b*. *athabascae*). Our study demonstrates how mitochondrial DNA can be applied to delineate the history of wildlife species and inform future conservation actions.

## Introduction

One of the most iconic species living in Yellowstone National Park (NP) is the American plains bison (*Bison bison bison*). American bison survived multiple historic and recent population bottlenecks due to habitat reduction, commercial hunting, and diseases from imported domestic livestock [1]. Populations undergoing major reductions in size with constrained areas of distribution are vulnerable to the effects of inbreeding and the loss of genetic diversity through genetic drift [2,3].

Yellowstone bison have existed on the same landscape for hundreds of years and there is no evidence of domestic cattle introgression [4,5,6]. The population reached its nadir in 1902, with as few as 22 indigenous animals remaining in the central area of the NP. As a result, managers reintroduced bison to the Lamar Valley in the northern region of the NP, including 18 females from the Pablo-Allard herd in northern Montana, three bison bulls from the Goodnight herd in Texas, and three calves from the indigenous bison in central Yellowstone [5,7]. The indigenous and introduced herds began commingling in 1915 and have intermixed seasonally to some extent ever since [5].

Today, the Yellowstone bison population occupies approximately 1 million acres of suitable habitat near the headwaters of the Yellowstone and Madison River watersheds [8]. The core habitat available to Yellowstone bison is protected by the management boundaries and conservation policies of the National Park Service. Additional suitable habitat for bison extends outside the park into Montana, but only constitutes less than 10% of the total conservation area [9]. Bison coexist with a full suite of native ungulates and predators, exposing them to competition for food, predation, and survival under substantial environmental extremes. Thus, Yellowstone bison have likely retained adaptive capabilities that may be diminished in other bison populations across North America that are managed like livestock [10].

Halbert et al. [11] evaluated 46 nuclear microsatellite loci from Yellowstone bison and found evidence of a moderately high level genetic diversity (0.626) and gene patterns indicating the existence of at least two subpopulations (the northern and the central herds) with limited gene flow between them. However, not much work has been conducted to describe genetic diversity based on the mitochondrial genome. Mitochondrial DNA (mtDNA) analyses provide insight into how historical events shaped and influenced population genetic diversity without the complicating issues of diploidy and recombination, inherent with the nuclear genome. Mitochondrial haplotype diversity is a valuable indicator of population health because mtDNA codes for genes that play a crucial part in ribosomal activity, cellular respiration, and energy production. The mitochondrial genome contains 13 protein-coding genes, as well as genes that code for the small and large rRNA subunits (12S and 16S respectively), and tRNAs. The mitochondrial genome is haploid and inherited only through the maternal lineage, making it easier to track populations without having to account for heterozygotes. In addition, mtDNA is more sensitive to inbreeding, loss of diversity, and genetic drift because only one parent plays a role in its transmission.

Ward et al. [12] analyzed 53 bison from across North America that had no evidence of cattle mtDNA and described eight unique bison haplotypes based on partial D-loop sequences from the mitochondrial genome. Analyzing DNA sequences from this highly variable 600 base pair region, the authors reported two haplotypes (which they named haplotypes 6 and 8) from the five Yellowstone bison analyzed. Gardipee [13] collected DNA samples from 153 Yellowstone bison and developed a method to distinguish between the two haplotypes previously described by Ward et al. [12] by sequencing a 470 base pair section of the D-loop control region.

Analyzing the complete mitochondrial genome, Douglas et al. [14] found 17 unique mtDNA haplotypes during a broad survey of plains bison (*B*. *b*. *bison*) and wood bison (*B*. *b*. *athabascae*). Wood bison are phenotypically distinct from plains bison and historically limited to Canada and the State of Alaska. Most of the 17 haplotypes came from animals in private herds, which have largely undocumented histories and cannot be traced back to a particular lineage. Notable exceptions are bHap2 which includes a bison at the National Bison Range in Montana, bHap10 includes a Fort Niobrara National Wildlife Refuge bison from Nebraska, bHap17 is from a Yellowstone NP bison, bHap13 and 16 from the Caprock Canyon State Park in Texas, and wHap14 and 15 from wood bison at Elk Island NP in Canada.

Based on the published sequences from Douglas et al. [14], Pringle [15] proposed that two non-synonymous point mutations in bison mitochondrial DNA cause significant impairment of mitochondrial oxidative phosphorylation (IMOP). One of these mutations causes an isoleucine to asparagine amino acid change in the ATP6 gene while the other is a valine to alanine change in the cytochrome b gene (S1 Table). His conclusions were deduced solely from comparative *in silico* analysis of homologous sequences in other mammals such as dogs and humans where similar mutations are known to cause a mitochondrial disease [16]. To our knowledge, no phenotype has ever been described to substantiate the detrimental effect of the IMOP mutations in bison.

The objective of our research was to better characterize and understand haplotype frequencies in Yellowstone bison. Previous attempts to delineate mitochondrial haplotype diversity in bison took a much broader approach, analyzing only a few bison for a single location across the United States, which likely resulted in significant local diversity going undetected. We evaluated the amount of genetic diversity in mtDNA in Yellowstone bison and developed a molecular method to test for differentiation between the two primary breeding herds (the northern and central herds). In addition, we assessed the overall genetic health of Yellowstone bison and analyzed the allegedly detrimental IMOP mutations to identify potential selective differences between bison that express IMOP mutations and bison that are wild type.

## Results

**Fig 1:**
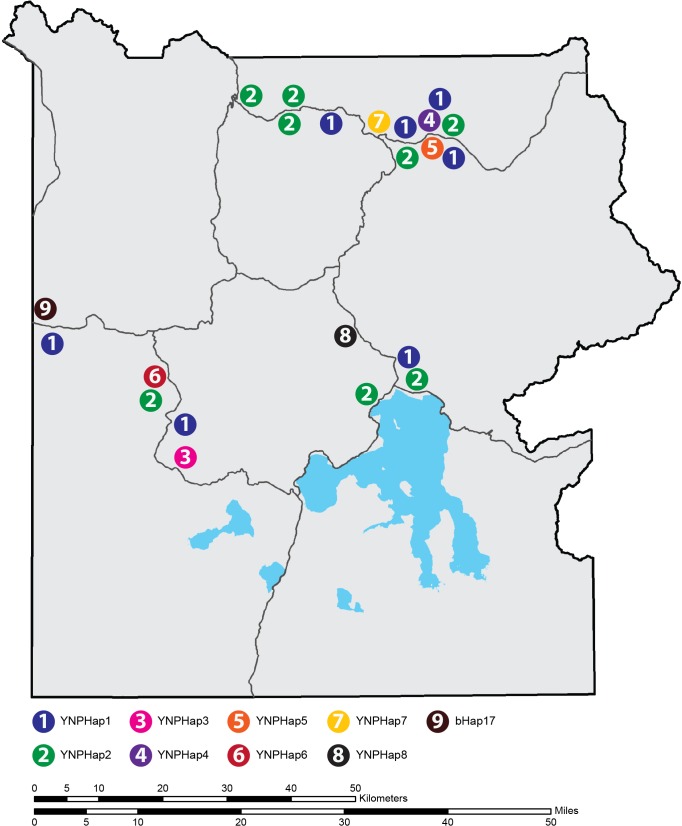
Mitochondrial haplotype distribution in Yellowstone National Park. The sampling location and haplotype identity of each Yellowstone bison in this study based on their association with either the northern or central herds. Three additional samples (Templeton, and two YNPHap2 bison) were collected from bison near the north boundary at a capture facility, but their movement histories are unknown, and they were omitted from this figure.

Ten different haplotypes were found in the 25 modern samples from Yellowstone bison (Table 1, Fig 1). Seven bison belonged in YNPHap1 and ten to YNPHap2. The rest of the haplotypes were unique to only a single animal sequenced in this study.

**Table 1:** Mitochondrial haplotype distribution in 25 bison associated with the northern or central herds in Yellowstone NP.

Haplotype	Northern Herd	Central Herd	Unknown	
YNPHap1	4	3		
YNPHap2	4	4	2	
YNPHap3	0	1		
YNPHap4	1	0		
YNPHap5	1	0		
YNPHap6	0	1		
YNPHap7	1	0		
YNPHap8	0	1	
bHap17	0	1		
Templeton	0	0	1	
Total	11	11	3	

Three samples were collected from bison near the north boundary at a capture facility, but their movement histories are unknown.

Haplotype diversity among all 25 modern Yellowstone bison was calculated as 0.7800 (+/- 0.0649) with a mean difference between the haplotypes of 0.00103. The AMOVA test for population subdivision between the northern and central herds yielded an F_ST_ value of -0.06 (p = 0.76). Arlequin is known to produce slightly negative F_ST_ values in cases where variation within the population is larger than variation between the groups that comprise the population [17]. In such cases, F_ST_ should be treated as zero [18,19,20]. Three of the 25 Yellowstone bison (Templeton, 5885, 5899) were sampled after they were removed from the population at the northern boundary of the NP, but they were not part of the telemetry study and were, therefore, excluded from the population subdivision analysis. bHap17 was sampled from the west boundary capture operation which is a migration path used only by the central herd.

While bison from the northern breeding group tend to remain in the northern area for their entire lives, bison born in central Yellowstone NP will either be year-round residents of the central range or migrate to the northern range to spend the winter. Observations over recent years indicate many bison from the central herd have emigrated to become residents in northern Yellowstone NP year-round [10,21] (Table 2).

**Table 2:** Mitochondrial haplotypes based on different life history strategies. Only the migration status of the 20 animals in the radio telemetry study were known and included.

Haplotypes	Year-round in central Yellowstone NP	Winter migrant to northern from central Yellowstone NP	Year-round in northern Yellowstone NP	Emigrant from central to northern Yellowstone NP	Total
YNPHap1	1	2	4	0	7
YNPHap2	3	1	1	3	8
YNPHap3	1	0	0	0	1
YNPHap4	0	0	0	1	1
YNPHap5	0	0	0	1	1
YNPHap6	0	1	0	0	1
YNPHap7	0	0	1	0	1
Total	5	4	6	5	20

**Fig 2:**
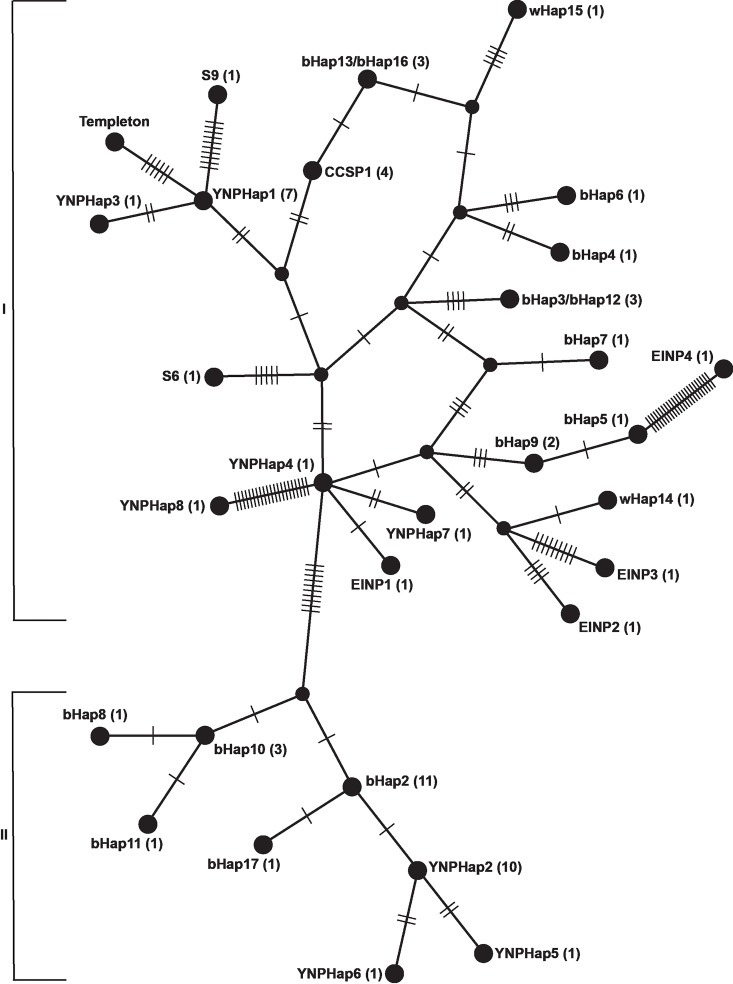
TCS network tree of North American bison, based on all polymorphic sites. Each dash represents one single nucleotide difference between two neighboring haplotypes. The numbers in the parentheses after the name of each haplotype denote the number of bison belonging to each haplotype. Roman numerals I and II represent the two clades in this analysis. Animals with a wHap and EINP prefix denote wood bison haplotypes, while YNPHap, Templeton, and bHap17 denote Yellowstone bison. S6 and S9 are two historic bison sampled in or near the modern day Yellowstone National Park.

**Fig 3:**
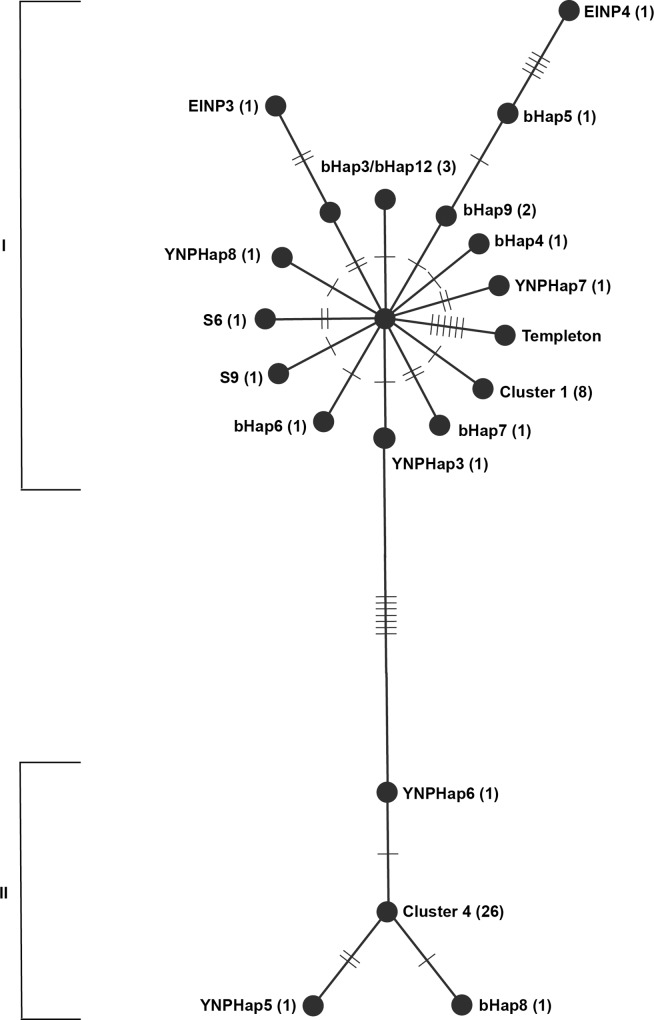
TCS network tree of North American bison based on non-synonymous sites. All polymorphic sites that are synonymous or not in protein-coding genes were disregarded for this tree. Each dash represents one single nucleotide difference between two neighboring haplotypes. The numbers in the parentheses after the name of each haplotype denote the number of bison belonging to each haplotype. Roman numerals I and II represent the two clades formed in this analysis.

The analyses of all 65 plains and wood bison from across North America based on the combined data from Douglas et al. [14] and this study revealed 30 different haplotypes with a total of 120 polymorphic sites. Forty-six of these sites were synonymous, 37 were non-synonymous, and 37 were polymorphic sites in tRNA- and rRNA-coding regions. The only genes with no nucleotide differences between the 65 bison were the ND4L protein-coding gene, and the tRNA-Val, tRNA-Gln, tRNA-Met, tRNA-Tyr, tRNA-His, tRNA-Ser, tRNA-Glu, and the tRNA-Pro genes. The Templeton-Crandall-Singh (TCS) network tree (Fig 2 and Fig 3) identified two distinct clades of haplotypes separated by 10 polymorphisms unique to every member of each clade. Due to the large constraint on the mitochondrial genome, non-synonymous mutations in protein-coding genes that cause a change in the amino acid sequence are deemed especially important. In an attempt to reduce the noise in the tree in Fig 2, and to see if the clear divide between the two clades is still supported, a second tree only considering the 37 non-synonymous mutations was created (Fig 3). This tree disregards all nucleotide differences in tRNA and rRNA genes, as well as all synonymous changes in the 13 protein-coding genes in the mitochondrial genome.

Some bison that formerly represented unique haplotypes collapsed into clusters that are compilations of haplotypes that were unique when all polymorphisms were taken into consideration, but lack any non-synonymous mutations to differentiate between them. Cluster 1 includes haplotypes CCSP1, bHap13, bHap16, and wHap15; Cluster 2 includes YNPHap1, YNPHap4, and EINP1; Cluster 3 includes EINP2, and wHap14; and Cluster 4 includes YNPHap2, bHap2, bHap10, bHap11, and bHap17. This TCS network further supports that the separation between the two clades is not just an artifact in the dataset (Fig 3).

Thirteen of the 25 Yellowstone bison belonging to YNPHap2, YNPHap5, YNPHap6, and bHap17 exhibited both alleged detrimental IMOP mutations, while the other 12 bison belonging to YNPHap1, YNPHap3, YNPHap4, YNPHap7, YNPHap8, and Templeton did not have the mutations. All animals exhibited either both or neither point mutations. Clades I and II align in such a way that all animals in Clade I that includes the indigenous Yellowstone bison are wild type, while all animals in Clade II that include the bison introduced in 1902 have both IMOP mutations (Fig 2).

## Discussion

The analysis of the mitochondrial genome of 25 Yellowstone bison yielded ten unique haplotypes, demonstrating high haplotype diversity in this population 0.78 (± 0.06). These haplotypes had little differentiation between them with the overall mean difference of 0.00103, which demonstrates the historic bottleneck and the subsequent process of increasing diversity due to the population boom and good management practices. High diversity is associated with greater population health and higher fitness in animals [22,23]. Similar results were found in a woodrat population in Texas where haplotype diversity was high (0.974 overall) but nucleotide diversity was low (0.008), suggestive of a period of low effective population size followed by rapid expansion [24].

Using the 22 Yellowstone bison sampled for this study where sampling locations were well documented, population subdivision was tested on a geographic scale, using the northern and central herds as the two hypothetical populations. This study did not find any evidence of population subdivision between the two herds based on mitochondrial DNA. Halbert et al. [11] found evidence for population subdivision and the existence of at least two breeding herds within the Yellowstone bison population based on STRUCTURE analysis [25] using 43 nuclear microsatellites, but reported similar F_ST_ values to ours (0.0321). The reason for the difference in the findings could be due to differences in the structure and function of the genomic regions analyzed, the differences in mutation rates, and the sensitivities of the statistical tests used.

When the 10 Yellowstone haplotypes were compared with the 20 other bison haplotypes from across the United States and Canada, there was a clear division between Clade I and II. The comparatively large genetic distance between the two clades– 10 single nucleotide polymorphisms in contrast to the smaller distance between internal nodes within each clade–shows the signature of a major historic event that explains the separation between the two. Clade I contains haplotypes that are more similar to the two historic bison (S6 and S9) that lived in or near modern day Yellowstone National Park, while Clade II consists of bison that are genetically more dissimilar to those. Clade II also includes animals that are known to have originated from the Pablo-Allard herd, such as the samples from the National Bison Range, Fort Niobrara National Wildlife Refuge, as well as the bison introduced to northern Yellowstone NP in 1902. Thus, we conclude that Clade I contains haplotypes that are more closely associated with the indigenous bison that lived in the area for hundreds of years while Clade II has haplotypes that resemble the bison introduced to the park from northern Montana in 1902.

Our findings indicate that no bison in Clade I have the mutations implicated in IMOPs, while both mutations were present in all bison in Clade II. Therefore, all the introduced bison from the Pablo-Allard herd and their descendants have these mutations. Comparing the frequency of IMOPs in 1902 with current frequencies enables a comparison of Yellowstone bison mtDNA haplotype frequencies over a span of 110 years. If these mutations truly cause a detrimental phenotype, as hypothesized by Pringle [15], a substantial reduction in the frequency of haplotypes with IMOPs would be expected due to strong negative selection. In 1902, there were a total of 10 indigenous females and 18 introduced females, while the 25 modern-day bison sampled are split with 13 animals with IMOPs and 12 that are wild type (Table 3). Because the descendants of the indigenous bison lacked the IMOP mutations (Clade I), and the descendants of the introduced animals possessed the mutations (Clade II), we can compare the frequency of 1902 introduced bison and the current bison that were found to carry the IMOP mutations, as well as the frequency of the 1902 indigenous bison and the current frequency of wild type bison. Due to the statistically non-significant change in haplotype frequencies in the Yellowstone population (p = 0.412) based on Fisher’s exact test and the lack of any kind of reported lesion or disease that affect a large proportion of Yellowstone bison, we did not find evidence to support Pringle’s hypothesis.

**Table 3:** Historic and present frequencies of IMOP mutations in Yellowstone bison.

	IMOPs	Wild type
Number of bison in 1902	18 (introduced females)	10 (indigenous females)
Frequency in 1902	0.643	0.357
Bison in current study	13	12
Current frequency	0.520	0.480

The present study has contributed eight new haplotypes to a large dataset with over a million base pairs of mitochondrial DNA sequence analyzed from bison across North America. These data have significant implications beyond the Yellowstone bison population. For example, the bison network (Fig 3) indicates that wood bison (*Bison bison athabascae*) are not a single monophyletic group. Approximately 6,600 plains bison from the Conrad herd were used to supplement the local population in Wood Bison NP in the 1920s [7]. Hybridization between plains and wood bison is well documented [26,27], which certainly confuses their current taxonomical status. Elk Island NP received 23 animals from Wood Bison NP in 1965. While Cluster 3 (Fig 3) represents a monophyletic group that is markedly different from the closest plains bison haplotype, others, such as wHap15, represent animals that may phenotypically look like wood bison but their mitochondrial DNA is closely related to plains bison. The closest haplotypes to wHap15 are bHap13/bHap16. These two haplotypes are from the descendants of the historical bison herd of Charles Goodnight which now constitutes the Caprock Canyon State Park herd in Texas. Historical documents show that Conrad purchased a bison heifer from Goodnight before he sold plains bison to the Canadian government that they used to supplement the bison herd in Wood Buffalo NP [7].

Mitochondrial DNA analysis can be used as an indicator for population loss because it is four times more sensitive to any reductions in the effective population size compared to the nuclear genome [28]. Sequencing and analyzing a large dataset, such as whole mitochondrial DNA from many animals in a wildlife population, can reveal various facets that are telling about the history and genetic health of that population. Therefore, a vast amount of new information can be used to improve the conservation and management of species at risk of population reduction or extinction. Advances in sequencing technologies have allowed us to sample more animals and sequence whole mitochondrial genomes to discover eight new haplotypes and provide a finer resolution than previous studies. Due to the high number of haplotypes identified relative to the number of individuals sampled in this study, a higher proportion of the Yellowstone population should be sampled to capture a more comprehensive array of haplotype diversity that exists in Yellowstone bison. Our analysis suggests that Yellowstone bison represent nearly half– 10 of 22 modern plains bison haplotypes–of all the known haplotypes in plains bison from recently sampled individuals.

These data could be used in the future to develop a mitochondrial DNA-based assay to screen Yellowstone bison to determine lineage. Based on our findings, sequencing a 1223 bp region using 18F and 18R primers from Douglas et al. [14] is sufficient to determine if a particular bison belongs to Clade I or Clade II. This sequence contains two of the 10 mutations separating the two clades which is enough to delineate whether the bison is a descendant of the indigenous or the introduced herd. We plan on sequencing more Yellowstone bison to develop a SNP-based assay to gain a more accurate picture of the genetic differentiation and migration patterns.

The status of the Yellowstone bison population based on our findings of high haplotype diversity and lack of population subdivision appears to be genetically healthy, especially for a population with a history of intensive management that included periods of extreme reductions in size. In recent years, as the number of bison has grown exponentially and more bison leave the park during the winter, culling of animals to control their abundance and distribution has become necessary. Our finding that there is no subdivision based on mtDNA support that Yellowstone bison can be managed–for mitochondrial haplotype diversity–as a single population with multiple breeding segments. Before new management standards and policies are defined for the Yellowstone bison population, additional studies involving population structure and genetic diversity based on both mtDNA and nuclear genetic diversity assessments need to be conducted.

## Methods

The U.S. National Park Service Institutional Animal Care and Use Committee has overseen and approved the use of bison in this study.

Approximately 30 adult female bison are fitted with radio telemetry collars each year in Yellowstone NP to obtain demographic and movement information [21,29]. During November 2011 to January 2012, tail hairs and blood was collected from 20 of these bison. The bison were chemically immobilized with Carfentanil and Xylazine following standard capture procedures periodically revised, reviewed, and endorsed by supervising veterinarians within the National Park Service and approved by park management. The National Park Service conducts reviews by an agency endorsed Institutional Animal Care and Use Committee which has reviewed anesthesia procedures conducted by the Yellowstone Bison Ecology and Management Program and has approved it as a monitoring and surveillance project. The IACUC reviews capture procedures every 3 years with the latest review completed in October of 2015. The blood samples were spotted on Whatman FTA cards and stored at room temperature. Bison were selected to represent four distinct survival strategies and movement patterns (Table 2), including year-round residents in northern Yellowstone NP (n = 6), year-round residents in central Yellowstone NP (n = 5), bison that emigrated from central to northern Yellowstone NP and remained for breeding (n = 5), and bison that migrated from central to northern Yellowstone NP during winter, but returned to central Yellowstone NP for breeding (n = 4).

This study focuses only on the protein-coding, tRNA and rRNA genes of the mitochondrial genome and excludes most of the D-loop control region due to difficulties in sequencing long mononucleotide runs and lack of known adaptive evolutionary function. mtDNA was extracted from these Yellowstone samples (haplotype ID: YNPHap n = 20) and sequenced with a 3130 Genetic Analyzer (Life Technologies). The mitochondrial genomes were amplified based on the PCR protocol described in Douglas et al. [14], and assembled using NCBI Reference Sequence NC_012346.1 [30]. The sequences were compared to previously published full mitochondrial haplotypes from Douglas et al. [14] (haplotype IDs: bHap for plains bison and wHap for wood bison, n = 31), with the exception of bPub1 which is a zoo animal of unknown lineage that was excluded from further analysis.

Illumina whole genome sequencing was conducted for 14 additional bison. Four of these animals were from Caprock Canyon State Park in Texas (CCSP, 5x coverage); four were wood bison from Elk Island NP in Canada (EINP, 5x coverage); four were additional Yellowstone animals that were collected from bison during winter removal operations (YNP1861, YNP5885, YNP5899, 10x coverage, and Templeton, 75x coverage); and two were historic museum specimens (S9 and S6, 10x coverage). The coverage values represent the average depth across the entire genome, mitochondrial reads are much more abundant due to the presence of hundreds of copies of the mitochondrial genome for each cell [31]. For modern samples used in this study, the mitochondrial coverage ranges from 86x-603x. S9 was collected from the Lamar Valley in northern Wyoming in 1856, and S6 was collected from southern Montana in 1886, near the northern boundary of Yellowstone NP [32] and the mitochondrial coverage for these historical samples was approximately 58x. There is limited information about the breeding ranges or movements of these 14 additional bison. Sequences were trimmed using FASTQ-MCF requiring a nucleotide quality score of more than 20 for each base and retaining only those reads that had a sequence length of more than 70 bases [33]. These filtered paired-end sequences were aligned to a previously published complete mitochondrial genome of a bison (YNP1586, GenBank ID: GU947004.1) using the default settings in the Burrows-Wheeler Alignment 0.6.2 software package (BWA-MEM) [34]. The resulting alignment files were sorted and indexed using SAMtools 0.1.18 [35]. Read group information was added using the AddOrRelpaceReadGroups option of PicardTools 1.7.1 (https://github.com/broadinstitute/picard/releases/tag/1.128). The Genome Analysis Toolkit 3.1.1 (GATK) [36] option RealignerTargetCreator was used to realign the mapped reads to account for INDEL shifted coordinates. Genetic variants, SNVs, and INDELs were identified against the mitochondrial bison sequence for each aligned sample and were filtered according to the GATK Best Practices recommendations [37,38]. These variants were placed into variant call formatted (VCF) files in order to make a consensus sequence for each sample using VCFtools package [39].

The older historic bison, S9 from the year 1856, shows increased frequency of C to T nucleotide substitutions, potentially due to de-amination well documented in historic samples [40] (S1 Table). However, all of these were unique to S9 only, meaning they only affect the length terminal node and not where the branch is located. Thus, these potentially de-aminated sites were presented in Fig 2 as they were sequenced, the only effect being the likely over-estimated genetic difference between S9 and the basal haplotype YNPHap1.

The notion that the sequences analyzed in this study were truly mitochondrial in origin and not NUMTs–inactive copies of pieces of mtDNA copied into the nuclear genome–is supported by the lack of frameshift and nonsense mutations [41] and a minimum coverage of 4x for any given base, without any sign of heteroplasmy. The mean length of numts are 100–300 bp [41], therefore primer sets amplifying 573–1223 bp fragments were used to avoid amplifying short nuclear copies during Sanger sequencing. Parts of mitochondrial sequences that had several SNPs close together in one, or a small subset of bison, have been checked using BLAST and yielded no suspicious nuclear sequences with a high identity score [42].

All the data from the 65 animals were compiled and aligned using ClustalW in MEGA version 6.0 [43]. The alignments were trimmed to the same length, 15,548 bp. Each polymorphic site was characterized based on whether it represented a synonymous or non-synonymous mutation or was in a non-coding region for further analysis (S1 Table).

Primers 18F (CTTTACCGCCATRGAACTAATCTT) and 18R (GTTCCTAAGACCAACGGATRA) were implemented from Douglas et al. [14] to amplify a region of the mitochondrial genome containing two SNPs that separate Clade I and Clade II. The two SNPs are both G/A transitions that cause non-synonymous changes in the ND4 gene, Ile10678Met and Ala11108Thr. Sequencing the 1223 bp region flanked by these primers can serve as a diagnostic test to determine clade identity in lieu of sequencing the entire mitochondrial genome.

Haplotype diversity was calculated for the Yellowstone bison as ${\hat{H}}_{e}=\frac{n}{n-1}(1-\sum_{i=1}^{n}{\hat{p}}_{i}{}^{2})$, where n is the number of bison and p is the frequency of each haplotype. Overall mean difference was determined by averaging the number of base substitutions per site over all sequences using the Maximum Composite Likelihood model in MEGA version 6.0 [44]. Population subdivision was calculated using Arlequin’s AMOVA feature to acquire the F_ST_ values based on the presence or absence of panmixia between the northern and central breeding herds. Phylogenetic networks were created using alignments imported in PopART v. 1.7 (http://popart.otago.ac.nz) and drawn as a TCS network using statistical parsimony (Fig 2 and Fig 3) [45,46]. A maximum likelihood tree with 500 bootstraps, under the model of gamma-distributed rate heterogeneity amongst sites and a proportion of invariant sites (G+I) was also created using MEGA version 6.0, using water buffalo (*Bubalus bubalis*) (GenBank ID: AY488491.1), yak (*Bos grunniens*) (AY684273.2), domestic cattle (*Bos taurus*) (GU947021.1), and European bison (*Bison bonasus*) (HQ223450.1), as the outgroups (S1 Fig).

## Supporting Information

S1 Fig Maximum likelihood tree showing all 30 bison mitochondrial haplotypes. The branch lengths depicted are not proportional to the actual genetic distance due to the high similarity of some neighboring haplotypes.
(TIF)

S1 Table Polymorphic sites in the bison mitochondrial genome. A breakdown of the most common variant of each of the 120 polymorphic sites in bison and the list of haplotypes that differ from it. Non-synonymous mutations are in red, synonymous mutations in green and non-coding tRNA or rRNA regions in black. The two hypothesized IMOP mutations are highlighted in yellow.
(XLSX)

S2 Table Sample origins and demographic information. Sample IDs and relating haplotypes for 25 modern Yellowstone NP bison from the study.
(XLSX)

S3 Table GenBank accession numbers for all previously unpublished haplotypes. (XLSX)
