# Supplementary figures and images for: Mitochondrial Genome Analysis Reveals Historical Lineages in Yellowstone Bison
Source: PLoS One. 2016 Nov 23;11(11):e0166081. doi: 10.1371/journal.pone.0166081 (PMC5120810; doi:10.1371/journal.pone.0166081)

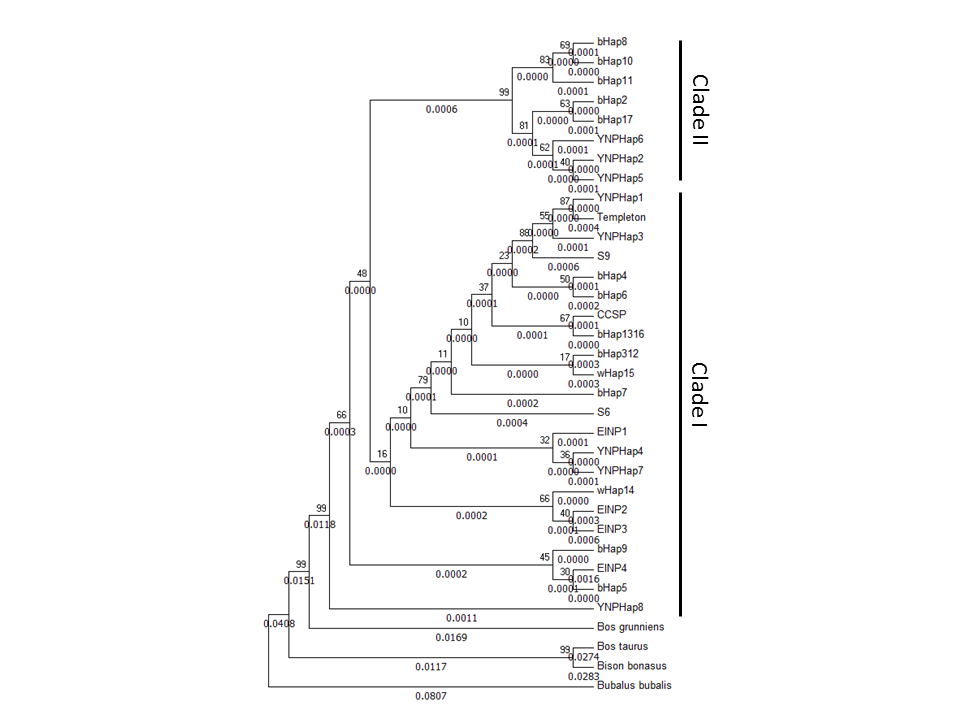

Supplement: S1 Fig — The branch lengths depicted are not proportional to the actual genetic distance due to the high similarity of some neighboring haplotypes. (TIF) [file pone.0166081.s001.tif]
